# Loss of the RNA-binding protein Rbm15 disrupts liver maturation in zebrafish

**DOI:** 10.1074/jbc.RA120.014080

**Published:** 2020-06-09

**Authors:** Liang Hu, Hongyan Li, Zhiping Chi, Jianbo He

**Affiliations:** Institute of Developmental Biology and Regenerative Medicine, Southwest University, Beibei, Chongqing, China

**Keywords:** RNA-binding motif protein 15 (Rbm15), liver organogenesis, liver maturation, mTORC1, hepatocyte nuclear factor 4alpha (Hnf4a), RNA-binding protein, gene regulation, post-transcriptional regulation, zebrafish hepatocyte nuclear factor 4 (HNF-4)

## Abstract

Liver organogenesis begins with hepatic precursors in the foregut endoderm, followed by hepatoblast specification, differentiation, outgrowth, and maturation for the formation of functional hepatocytes. Although several signaling pathways and critical factors that regulate liver specification, differentiation, and proliferation have been identified, little is known about how liver maturation is regulated. Here, we used a screen for mutations affecting liver development in zebrafish and identified a *cq96* mutant that exhibits a specific defect in liver maturation. Results from positional cloning revealed that *cq96* encodes an RNA-binding protein, Rbm15, which is an evolutionarily conserved Spen family protein and known to play a crucial role in RNA m6A modification, nuclear export, and alternative splicing. However, a function of Rbm15 in embryonic liver development has not been reported. We found that *Rbm15* is specifically expressed in the liver after its differentiation. CRISPR/Cas9-mediated loss of *rbm15* repressed hepatic maturation, but did not affect hepatoblast specification, differentiation, and hepatocyte proliferation and apoptosis. Additional experiments disclosed that the mTOR complex 1 (mTORC1) pathway is highly activated in *rbm15*-deficient hepatocytes. Moreover, rapamycin treatment partially restored normal hepatic gene expression as well as the nuclear location of the transcription factor Hnf4a. Taken together, these results reveal an unexpected role of Rbm15 in liver maturation.

The liver is the biggest digestive organ of the body and plays a central role in metabolism and systemic homeostasis. It mainly consists of hepatocytes, stellate cells, macrophages, sinusoidal endothelial cells, and cholangiocytes (1). The hepatocyte is the most populous cell of the liver; nearly 80% of hepatic cells are hepatocytes. It undertakes multiple physiological functions, such as bile secretion, glycol metabolism, lipid storage, protein secretion, and detoxification (2). Therefore, it is crucial to understand the mechanisms underlying liver development using animal models. Although the molecular regulation on liver organogenesis is starting to be unraveled, the critical factors to regulate liver development specifically have not been completely known.

In zebrafish, hepatogenesis can be divided into two steps. In the first stage, mesoderm around foregut endoderm induces hepatic precursor's proliferation and migration out from the gut endoderm and then specification to hepatoblast. Multiple layers of hepatoblast and the surrounding septum transversum form the liver bud. This progress lasts from 24 h post-fertilization (hpf) to 34 hpf (3). Mesoderm-derived FGF, WNT, and BMP signaling orchestrate this progress (4–6). Many transcription factors, such as GATA6, Hhex, and Foxas, are implicated in liver bud formation and hepatoblast specification (7–9). In the second stage, hepatoblast proliferates, differentiates, and maturates into hepatocytes and cholangiocytes, eventually forming a functional liver (2). In this progress, Wnt signaling is the key regulator during liver formation via increasing cell proliferation and repressing apoptosis (10, 11). The Hippo/YAP pathway is crucial for liver size control; hyper-YAP1 activity in the liver stimulates hepatocyte and cholangiocyte specification, whereas loss of YAP1 decreases hepatocyte survival and impairs biliary duct morphogenesis (12). Notch signaling and *esr2b* are also involved in hepatocyte differentiation and bile duct formation (13, 14). Numerous transcription factors govern cell fate choices. Hnf4a, Prox1, Hnf1a, and c/EBPa are important for hepatocyte differentiation and hepatic gene expression (15–18). Hnf1b and Hnf6 are responsible for bile duct formation (18). The mTOR pathway is also involved in liver development (19). Although many mechanisms underlying the hepatic fate decisions *in vivo* and *in vitro* have been reported, the key transcriptional factors that regulate hepatocyte maturation have been addressed less.

Rbm15 is an evolutionarily conserved Spen family protein involved in cell fate decisions. It can bind to RNA and recruit other proteins to specific binding sites to regulate post-transcriptional modification (20). In mice, RBM15 regulates megakaryocyte terminal differentiation by affecting alternative RNA splicing of a group of genes such as *Gata1*, *C-mpl*, *Runx1*, and *Tal1*, which are important for megakaryopoiesis (21). RBM15 can bind to specific sites in the intron of pre-mRNA, recruit SF3B1 to the splicing region, and facilitate alternative RNA splicing in different developmental stages (21). RBM15 can affect promoter activity of Notch target genes, such as *Hes1*, in a cell-specific manner. It can repress the expression of Notch-induced gene *Hes1* in nonhematopoietic cells but enhance the *Hes1* mRNA level in hematopoietic cell lines (22). RBM15 can also affect hematopoietic stem cell and megakaryocyte development via regulating c-*myc* expression (23). m6A is the most widespread RNA modification; the establishment of m6A relies on RBM15 (24, 25). RBM15 is also involved in RNA nuclear export (26). However, the role of Rbm15 in liver development is unknown.

Zebrafish has been developed into a popular model organism in recent years because of advantages of their transparent embryos developing outside of the mother, allowing constant visualization, and 69% of all zebrafish genes have a clear human orthologue (27). High genetic conservation among vertebrates makes zebrafish an excellent model system to study liver development and disease (27, 28). Here we used chemical mutagenesis to screen genes involved in liver development and got a specific liver maturation mutant, *cq96*. The mutant *cq96* revealed normal liver specification, differentiation, and outgrowth, but the liver maturation was repressed. The development of the pancreas and intestine showed normal in *cq96* mutant. Positional cloning and knockout experiments confirmed this mutation site located in gene *rbm15*. Deficiency of Rbm15 leads to up-regulation of the activity of mTORC1 signaling, and inhibition of the mTORC1 pathway by rapamycin can rescue liver maturation defect. Furthermore, the treatment of rapamycin enhanced the nuclear import of Hnf4a and restored hepatic gene expression. Our data revealed an unexpected function of *rbm15* in liver development in zebrafish.

## Results

### The cq96 mutant confers a liver maturation defect

**Figure 1.**
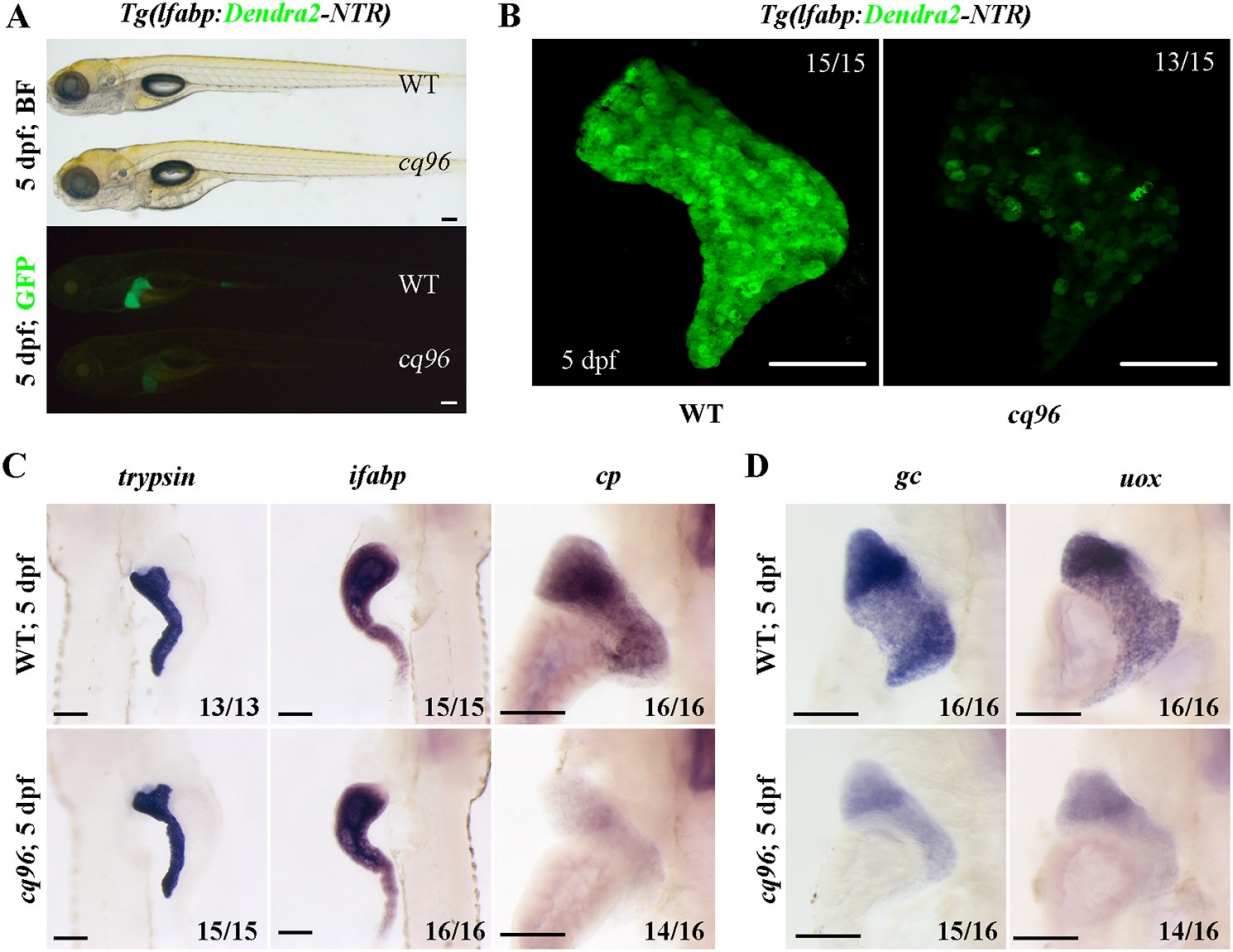
**Zebrafish *cq96* mutants display aberrant liver maturation.** The liver was labeled by *Tg(lfabp:Dendra2-NTR)^cq1^*. *A*, bright field and green fluorescence image of WT and *cq96* mutant at 5 dpf showing the morphology and liver fluorescence expression. *B*, confocal images of WT and *cq96* at 5 dpf revealing the liver size and morphology. *C*, whole-mount *in situ* hybridization (WISH) results showing the expressions of *trypsin*, *ifabp,* and *cp* in WT and *cq96* mutant at 5 dpf. *D*, evaluating the liver function by WISH results of *uox* and *gc* in WT and *cq96* mutant at 5 dpf. *BF*, bright field. *Numbers* indicate the proportion of larvae exhibiting the expression shown. *Scale bars*, 100 μm.

We have screened mutagenized zebrafish larvae using transgenic line *Tg(lfabp:Dendra2-NTR)* (29) and identified the *cq96* mutant. At 5 dpf, this mutant showed normal body morphology but weaker Dendra2 expression (Fig. 1*A*). The confocal image exhibited relatively normal liver size but heterogeneous Dendra2 expression in the mutant (Fig. 1*B*). The expressions of digestive organ–specific markers *trypsin* (exocrine pancreas) and *ifabp* (intestine) were normal, but the hepatocyte-specific gene *cp* was repressed (Fig. 1*C*). The expressions of functional markers of hepatocytes such as *uox*, *gc,* and *gys2* were repressed in the mutant (Fig. 1*D* and Fig. S1 (*A* and *B*)). These data indicate that the *cq96* mutant specifically affects liver development and especially regulates liver maturation.

### Zebrafish cq96 mutation site locates in gene rbm15

**Figure 2.**
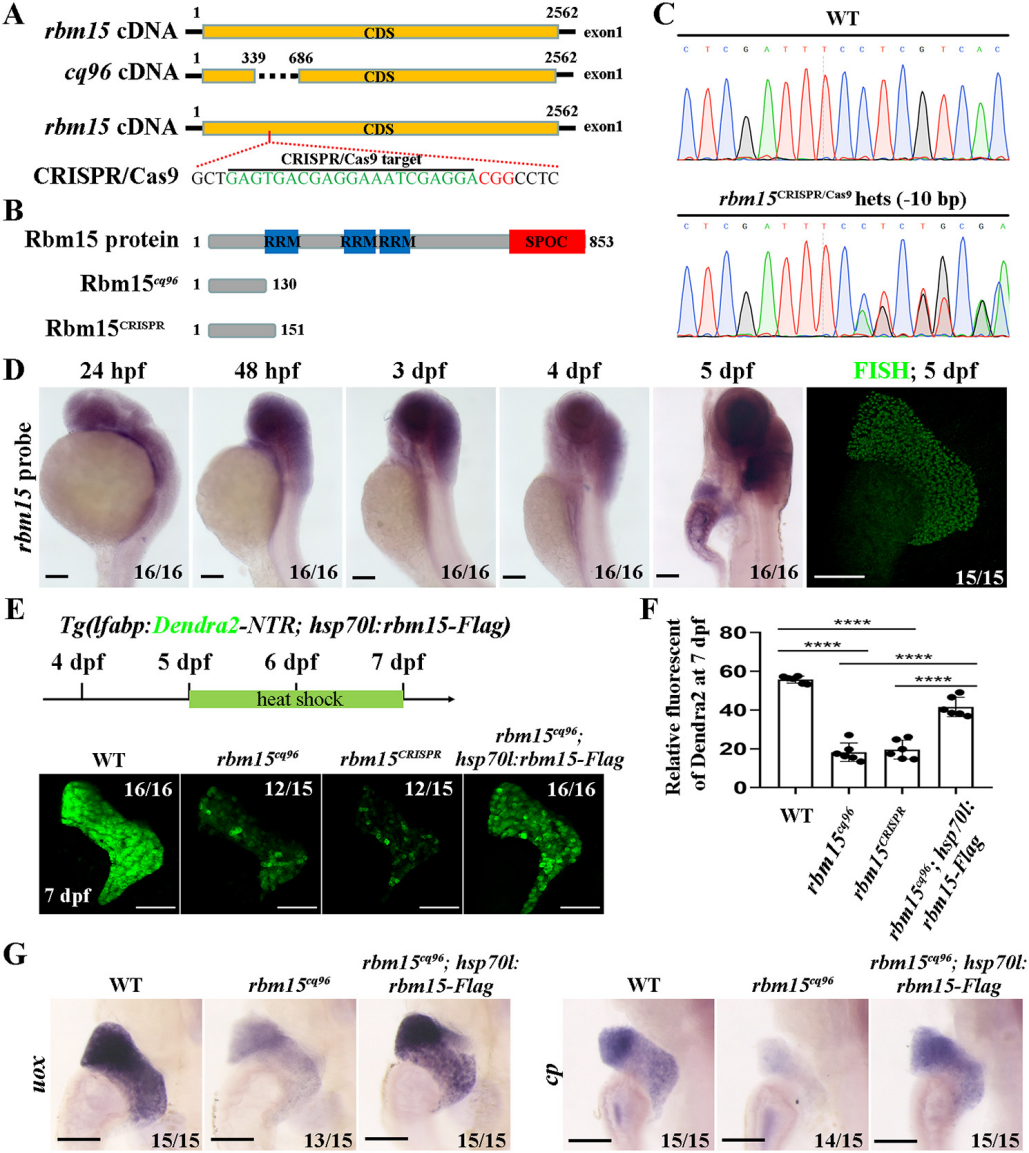
**The mutation gene in *cq96* is *rbm15.*** *A* and *B*, schematic drawing of the *rbm15* locus, *cq96*, CRISPR/Cas9 targeting site (*green*), and the PAM sequence (*red*). *C*, genomic sequencing result of *rbm15* mutation derived from the CRISPR/Cas9 knockout experiment. *D*, WISH and fluorescence *in situ* hybridization (*FISH*) results showing the expression pattern of *rbm15* from 24 hpf to 5 dpf. *E*, heat shock transgenic line *Tg(hsp70l:rbm15-Flag^cq97^)* rescues liver defects of *cq96*; confocal images show the expression of Dendra2. *F*, the quantification of fluorescent intensity of Dendra2 in the liver. *G*, evaluating the rescue effects by detecting the expression of liver functional genes *uox* and *gc* via WISH. *Numbers* indicate the proportion of larvae exhibiting the expression shown. *Asterisks* indicate statistical significance: ****, *p* < 0.0001. *Scale bars*, 100 μm; *error bars*, S.D.

To determine the target gene of the *cq96* mutant, we performed genome mapping and placed the *cq96* mutation site locus to gene *rbm15*. The genomic sequencing result showed that there are 347 bp deleted in *rbm15* exon 1, leading to translation into a truncated peptide (Fig. 2, *A* and *B*). To further confirm that *rbm15* affects liver maturation, we generated a new *rbm15* mutant by CRISPR/Cas9 that resembled the *cq96* phenotype (Fig. 2, *A–F*). These results revealed that the mutation gene of *cq96* is *rbm15*. Furthermore, *rbm15* expressed in the liver region from 4 dpf and much stronger at 5 dpf (Fig. 2*D*). The weak expressions of Dendra2 and hepatic maturation markers such as *uox* and *cp* in *cq96* mutant can be rescued by using Rbm15 overexpression line *Tg(hsp70l:rbm15-Flag^cq97^)* (Fig. 2, *E–G*). These results suggested that the mutation gene in *cq96* is *rbm15.*

### Zebrafish cq96 mutant affects liver maturation but not hepatoblast specification

**Figure 3.**
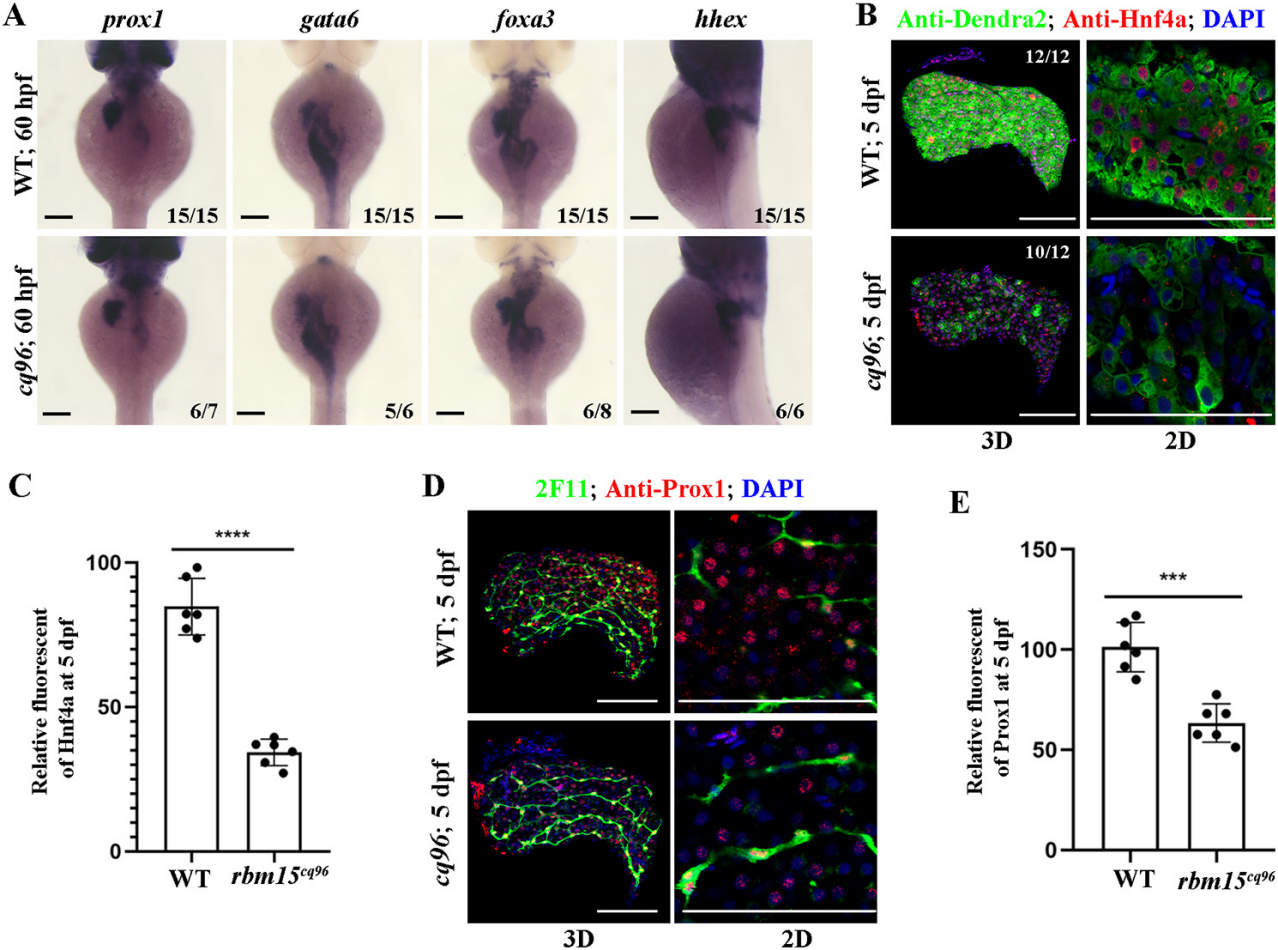
**Hepatoblast specification is normal in the *cq96* mutant.** *A*, WISH results of *gata6*, *prox1*, *foxa3*, and *hhex* at 60 hpf revealing hepatic formation and specification in *cq96* and WT. *B*, confocal images showing the antibody staining of Hnf4a and Dendra2 at 5 dpf in *cq96* mutant. *C*, the quantification of Hnf4a fluorescent intensity in WT and *cq96*. *D*, confocal images showing the antibody staining of Prox1 and 2F11 in WT and mutant at 5 dpf. *E*, the quantification of Prox1 fluorescent intensity in WT and *cq96*. *Numbers* indicate the proportion of larvae exhibiting the expression shown. *Asterisks* indicate statistical significance: ***, *p* < 0.001; ****, *p* < 0.0001. *Scale bars*, 100 μm; *error bars*, S.D.

To evaluate hepatoblast specification in the *rbm15* mutant, we assayed the expression of transcriptional factors important for hepatoblast formation and specification at 60 hpf. Interestingly, the expressions of *prox1*, *gata6*, *hhex*, and *foxa3* were not greatly different between mutants and siblings (Fig. 3*A*). This means that liver specification was normal in the *cq96* mutant. Then we performed antibody staining for hepatic factors Hnf4a and Prox1 at 5 dpf. The nuclear signal for Hnf4a and Prox1 was much weaker compared with siblings (Fig. 3, *B–E*). These results suggest that the *cq96* mutant has liver maturation rather than liver specification defects.

### Loss of Rbm15 confers normal hepatic proliferation and apoptosis

**Figure 4.**
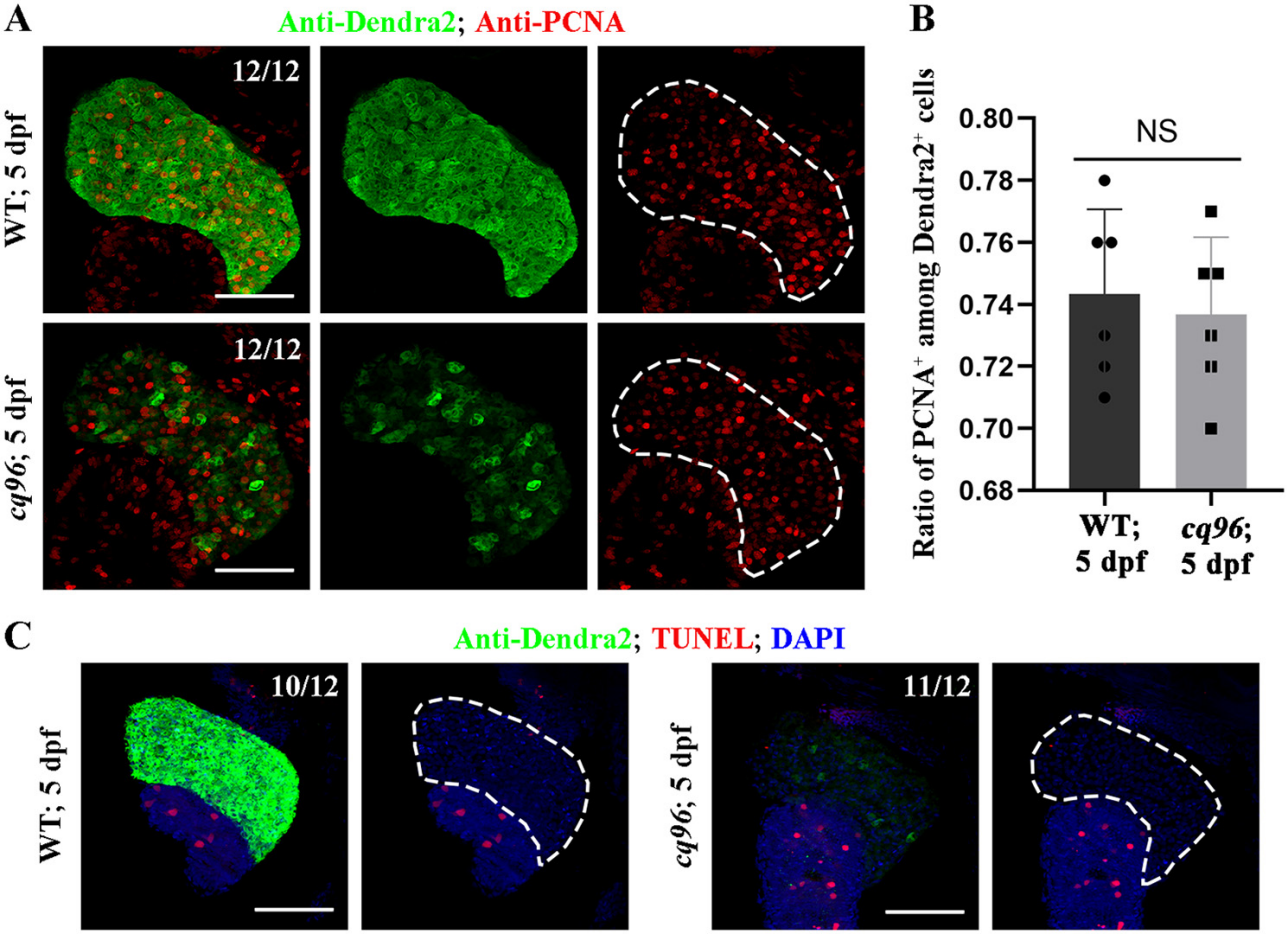
**Loss of Rbm15 has no effect on hepatic proliferation and apoptosis.** *A*, antibody staining results of PCNA and Dendra2 showing hepatocytes outside of the G_0_ phase at 5 dpf. *B*, quantification of the percentage of the PCNA^+^ among Dendra2^+^ cells at 5 dpf in WT and *rbm15*^−/−^. TUNEL assay images showing the apoptosis of liver in WT and *rbm15*^−/−^ at 5 dpf. *Numbers* indicate the proportion of larvae exhibiting the expression shown. *NS*, not significantly different; *scale bars*, 100 μm; *error bars*, S.D.

To investigate the underlying mechanism of liver development defect in *cq96* mutant, we performed antibody staining for PCNA, which labeled cells outside of the G_0_ phase. We detected a similar ratio of PCNA and Dendra2 double-positive cells in siblings and mutants (Fig. 4, *A* and *B*). To assess whether cell death contributes to liver development defect in *cq96* mutant, we performed a transferase-mediated dUTP nick-end labeling (TUNEL) assay on WT and *cq96* at 5 dpf. Larval hepatocytes presented a low apoptotic index at 5 dpf, which was unchanged in *cq96* mutant (Fig. 4*C*). Therefore, liver development defect in the *cq96* mutant does not appear to be due to defective cell proliferation and apoptosis.

### Inhibiting mTORC1 signaling pathway partially rescues the phenotype of cq96 mutant

**Figure 5.**
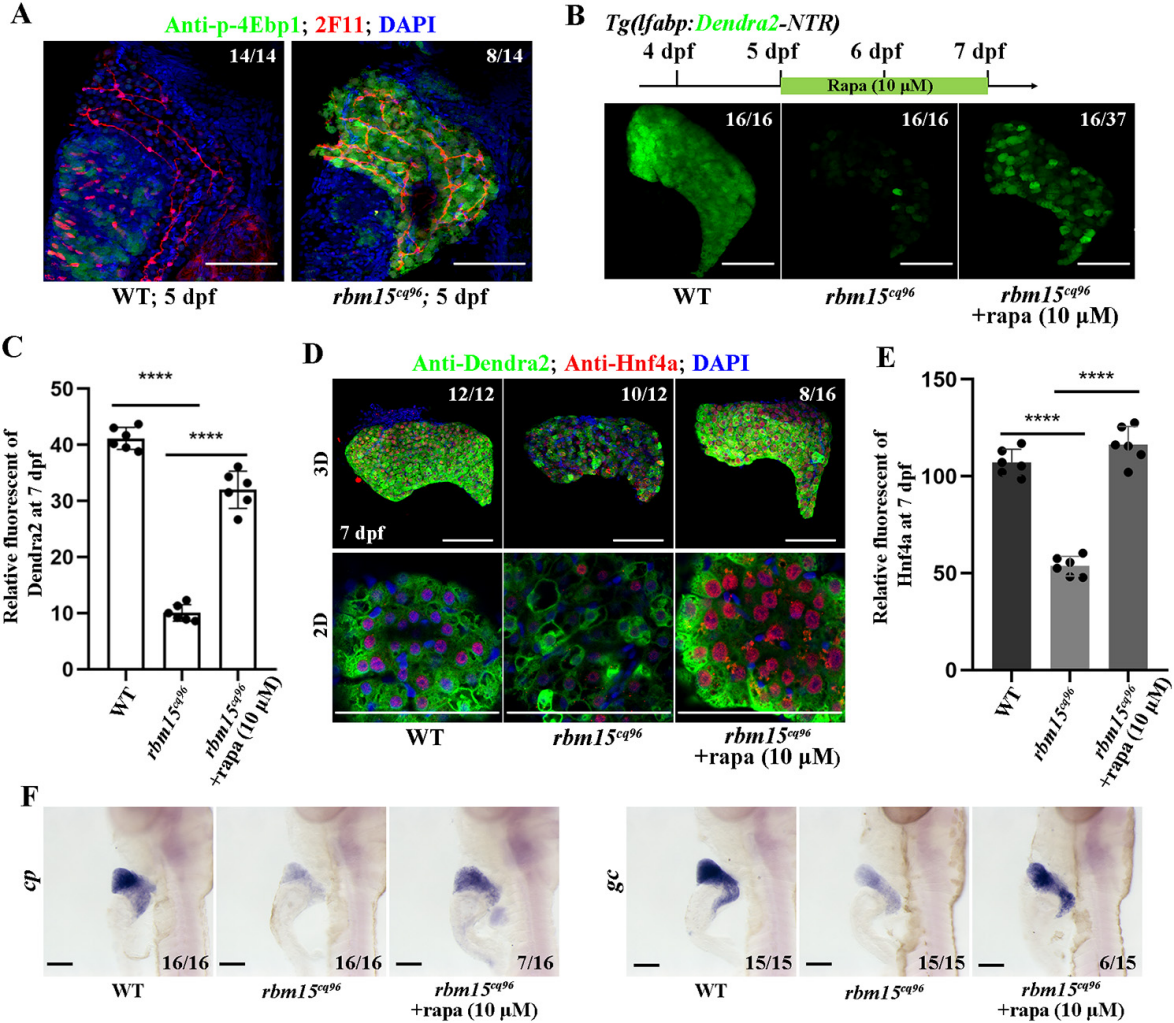
**Inhibiting the mTORC1 pathway partially rescues the liver maturation defects in *rbm15^cq96^*.** *A*, confocal images (*3D*) showing the antibody staining of p-4Ebp1 and 2F11 to evaluate the activity of mTORC1 signal in hepatocytes at 5 dpf. *B*, rapamycin treatment and confocal images reflecting the rescue efficiency of rapamycin treatment. *C*, quantification of Dendra2 fluorescent intensity in WT, *cq96*, and rapamycin treatment. *D*, antibody staining results of Hnf4a and Dendra2 showing that rapamycin treatment affects the cellular and protein levels of Dnedra2 and Hnf4a. *E*, the quantification of Hnf4a fluorescent intensity in WT, *cq96*, and rapamycin treatment. *F*, WISH images showing the expression patterns of *cp* and *gc* after rapamycin treatment. *rapa*, rapamycin. The *numbers* indicate the proportion of larvae exhibiting the expression shown. *Asterisks* indicate statistical significance: ****, *p* < 0.0001. *Scale bars*, 100 μm; *error bars*, S.D.

Hyper- and hypoactivated mTORC1 pathway will impair normal liver development (30, 31). To explore whether the loss of *rbm15* will affect the mTORC1 signaling or not, we performed antibody staining to check the expression level of p-4Ebp1, which indicates the activity of mTORC1 signaling, and found that the *rbm15*^−/−^ mutant larvae showed high p-4Ebp1 level in the liver (Fig. 5*A*). This indicates that the mTORC1 pathway was hyperactivated in the *rbm15*^−/−^ liver. To further confirm that the liver maturation defect in the *rbm15* mutant was caused by mTORC1 activation, we used 10 μm rapamycin to inhibit mTORC1 from 5 to 7 dpf. mTORC1 inhibition can partially rescue the developmental liver defect (Fig. 5 (*B* and *C*) and Fig. S2*A*). After rapamycin treatment, the protein level of Hnf4a in mutant hepatocyte was rescued (Fig. 5, *D* and *E*), but the mRNA level of *hnf4a* showed no big difference between WT, *rbm15*^−/−^, and sample groups (Fig. S2*B*). Furthermore, the expressions of hepatocyte-specific genes such as *cp* and *gc* were also restored after rapamycin treatment (Fig. 5*F*). These results indicate that inhibition of mTORC1 can partially rescue liver maturation defects in *rbm15* mutant.

## Discussion

We have described a liver developmental mutant caused by *rbm15* mutation, and inhibition of mTORC1 was an efficient strategy to rescue hepatic maturation defects. We showed that loss of Rbm15 specifically affected hepatic maturation, but not the developmental progress of intestine, pancreas. The mutant liver exhibited relative normal proliferation and apoptosis but weak hepatic gene expression. The two important hepatic transcriptional factors Hnf4a and Prox1 exhibited weak nuclear location, which could explain why *rbm15* mutant showed hepatic maturation defects to some degree. Liver failure caused by *rbm15* mutation also showed abnormal mTORC1 activation. Inhibition of mTORC1 can partially recover hepatic gene expression; this progress may rely on the enhancement of Hnf4a nuclear import.

Rbm15 is an important post-transcriptional regulator involved in RNA nuclear export, m6A modification, and alternative splicing (21, 25, 26). It is indispensable for megakaryocyte differentiation, and *rbm15* defect can induce acute megakaryoblast leukemia (20). mTORC1 acts as a metabolic regulator important for cell growth and differentiation (19). Abnormal mTORC1 activation is associated with liver developmental defects and enhancement of liver damage (30, 32). Our finding first reveals that *rbm15* is essential for hepatic maturation and that loss of *rbm15*-induced liver developmental failure partially depends on aberrant high mTORC1 activation.

We point out the importance of *rbm15* in hepatic maturation, but we still do not know the target genes of *rbm15*. The relationship between *rbm15* deficiency and high mTORC1 activity is still a mystery. RNA immunoprecipitation sequencing experiments will be indispensable to further answer these questions.

## Experimental procedures

### Ethics statement

All experimental protocols were approved by the Institute of Developmental Biology and Regenerative Medicine, Southwest University (Chongqing, China), and the methods were carried out in accordance with the approved guidelines. The zebrafish facility and study were approved by the Institutional Review Board of Southwest University (Chongqing, China). Zebrafish were maintained in accordance with the Guidelines of Experimental Animal Welfare from the Ministry of Science and Technology of the People's Republic of China (2006) and the Institutional Animal Care and Use Committee protocols from Southwest University (2007).

### Zebrafish lines

Zebrafish (*Danio rerio*) AB strain-derived *Tg(lfabp:Dendra2-NTR)^cq1^* was used as WT, and *rbm15^cq96^* mutant was generated by ENU treatment. The IND line was used for mapping. These zebrafish lines were raised under standard conditions, and embryos/larvae for the experiment were treated with 0.003% PTU (Sigma) from 24 hpf.

### CRISPR/Cas9-targeted rbm15 knockout

The CRISPR/Cas9 was carried out essentially as reported previously (33). The sequence for CRISPR RNA is shown in Fig. 2*A*. We used the following primers to identify the genotype of mutant: forward primer, 5′-GAATTCTGGCGGAGGAAGCA-3′; reverse primer, 5′-AAGCCGACCCAGTGCTAAC-3′.

### Whole-mount in situ hybridization and fluorescent in situ hybridization

Whole-mount *in situ* hybridization and fluorescent *in situ* hybridization were based on a previous report (29) using antisense probes for *hhex*, *gata6*, *foxa3*, *prox1*, *uox*, *gc*, *cp*, *hnf4a*, and *rbm15*. Primers used for amplifying the *rbm15* probe were 5′-GAGGCAGTTTACTTGAACAG-3′ (forward primer) and 5′-AAGCCGACCCAGTGCTAAC-3′ (reverse primer).

### Antibody staining and TUNEL assay

Antibody staining and TUNEL assay were performed as described previously (29). The following antibodies were used: antibodies against Dendra2 (1:1000; AB821, Evrogen, Moscow, Russia), phospho-4E-BP1 (Thr-37/46) (1:500; catalog no. 2855, Cell Signaling), Hnf4a (1:200; sc-6556, Santa Cruz Biotechnology, Inc.), Prox1 (1:500; ab5475, Chemicon), 2F11 (1:1000; ab71826, Abcam, Cambridge, MA), and PCNA (1:1000; SAB2701819, Sigma).

### Generation of transgenic line for rescue experiments

Full-length *rbm15* cDNA was amplified by PrimeSTAR HS DNA Polymerase (Takara) and cloned into *pBluescript* vector. The full-length *rbm15*-*Flag* CDS was driven by *hsp70l* promoter, and the plasmid was injected into AB strain embryos to generate the transgenic line *Tg(hsp70l:rbm15-Flag)^cq97^*. Positive embryos showed cerulean expression in the eyes of the offspring.

### Rapamycin treatment and heat shock

Embryos were treated with 10 μm rapamycin (Sangon Biotech, Shanghai, China) in PTU egg water from 5 to 7 dpf and replaced rapamycin solution every 24 h. The control group was treated with 0.2% DMSO. To induce *rbm15* overexpression from *Tg(hsp70l:rbm15-Flag)^cq97^*, larvae were placed in egg water and then incubated in a 38.5 °C water bath for 30 min once a day from 5 to 7 dpf.

### Data collection and analysis

All images were taken on a SteREO DiscoveryV20 microscope (Carl Zeiss, Germany) and LSM880 confocal microscope (Carl Zeiss). The intensities of fluorescent images were measured with ImageJ. The statistical analysis were performed with GraphPad Prism 8. Variation of individual data points was represented in S.D.

## Data availability

All the data are contained within the article.

## Supplementary Material

Supporting Information
