# Supplementary material for: Loss of the RNA-binding protein Rbm15 disrupts liver maturation in zebrafish
Source: J Biol Chem. 2020 Jun 9;295(33):11466–72. doi: 10.1074/jbc.RA120.014080 (PMC7450140; doi:10.1074/jbc.RA120.014080)
Supplement: Supporting Information [file supp_295_33_11466__index.html]

Loss of the RNA-binding protein Rbm15 disrupts liver maturation in zebrafish — Rbm15 regulates liver maturation — Loss of the RNA-binding protein Rbm15 disrupts liver maturation in zebrafish — Rbm15 regulates liver maturation — Supporting Information 

# Loss of the RNA-binding protein Rbm15 disrupts liver maturation in zebrafish

## Supporting Information

- Supporting Information (to be published online) - Supporting information
