# Supplementary material for: Loss of the RNA-binding protein Rbm15 disrupts liver maturation in zebrafish
Source: J Biol Chem. 2020 Jun 9;295(33):11466–72. doi: 10.1074/jbc.RA120.014080 (PMC7450140; doi:10.1074/jbc.RA120.014080)
Supplement: Supporting Information [file supp_RA120.014080_160437_2_supp_544258_qbmg6g.pdf]

Supplemental Data

Loss of the RNA-binding protein Rbm15 disrupts liver maturation in zebrafish

Liang Hu[1], Hongyan Li[1], Zhiping Chi[1], and Jianbo He[1]*

Supplementary Data:
Supplementary Figures: Figure S1 and S2

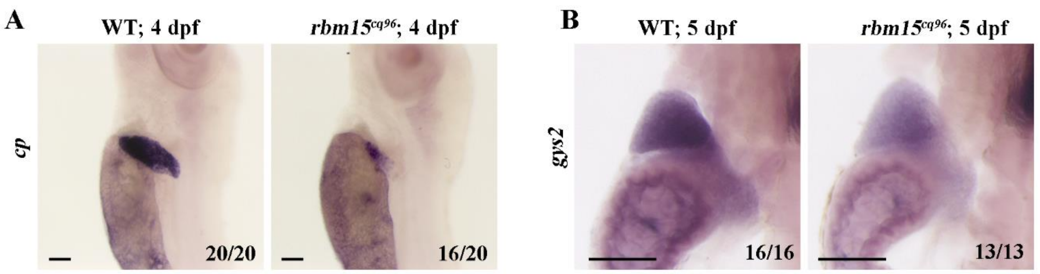

Figure S1

Supplemental Figure S1. **WISH result of *cp* and *gys2* in liver development. A.** WISH result of *cp* showing liver development of *rbm15*^{cq96} at 4 dpf. **B.** WISH result of *gys2* at 5 dpf revealing glycogenesis of *rbm15*^{cq96}. WT: wide type. Numbers indicate the proportion of larvae exhibiting the expression shown. Scale bars: 100 µm.

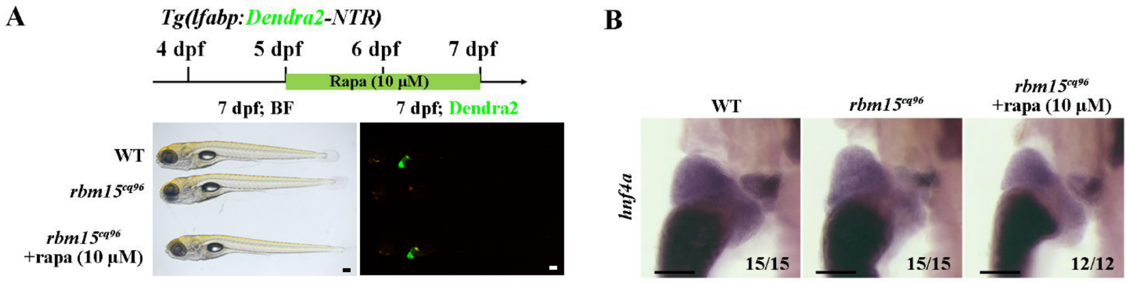

Figure S2

Supplemental Figure S2. **Rescue result of rapamycin treatment. A.** Rapamycin treatment strategy, and fluorescence images at 7 dpf. **B.** WISH result of *hnf4a* after rapamycin treatment showing the effect of rapamycin on the transcription level of *hnf4a*. BF: bright field. Numbers indicate the proportion of larvae exhibiting the expression shown. Rapa: rapamycin. Scale bars: 100 μm.
